# Genome Survey Sequencing of an Iconic ‘Trophy’ Sportfish, the Roosterfish *Nematistius pectoralis*: Genome Size, Repetitive Elements, Nuclear RNA Gene Operon, and Microsatellite Discovery

**DOI:** 10.3390/genes12111710

**Published:** 2021-10-27

**Authors:** J. Antonio Baeza, José Luis Molina-Quirós, Sebastián Hernández-Muñoz

**Affiliations:** 1 Department of Biological Sciences, Clemson University, Clemson, SC 29631, USA; 2 Departamento de Biología Marina, Universidad Católica del Norte, Larrondo 1281, Coquimbo, Chile; 3 Smithsonian Marine Station at Fort Pierce, Smithsonian Institution, Fort Pierce, FL 34949, USA; 4 Biomolecular Laboratory, Center for International Programs, Universidad Veritas, Zapote, San José 10105, Costa Rica; joseluismolina1993@gmail.com (J.L.M.-Q.); shernandez@veritas.cr (S.H.-M.); 5 Sala de Colecciones, Facultad de Ciencias del Mar, Universidad Católica del Norte, Larrondo 1281, Coquimbo, Chile

**Keywords:** genome skimming, low-pass genome sequencing, genomic resources

## Abstract

The ‘Pez Gallo’ or the Roosterfish, *Nematistius pectoralis*, is an ecologically relevant species in the shallow water soft-bottom environments and a target of a most lucrative recreational sport fishery in the Central Eastern Pacific Ocean. According to the International Union for Conservation of Nature, *N. pectoralis* is assessed globally as Data Deficient. Using low-coverage short Illumina 300 bp pair-end reads sequencing, this study reports, for the first time, the genome size, single/low-copy genome content, and nuclear repetitive elements, including the 45S rRNA DNA operon and microsatellites, in *N. pectoralis*. The haploid genome size estimated using a *k*-mer approach was 816.04 Mbp, which is within the range previously reported for other representatives of the Carangiformes order. Single/low-copy genome content (63%) was relatively high. A large portion of repetitive sequences could not be assigned to the known repeat element families. Considering only annotated repetitive elements, the most common were classified as Satellite DNA which were considerably more abundant than Class I-Long Interspersed Nuclear Elements and Class I-LTR Retroviral elements. The nuclear ribosomal operon in *N. pectoralis* consists of, in the following order: a 5′ ETS (length = 948 bp), ssrDNA (1835 bp), ITS1 (724 bp), a 5.8S rDNA (158 bp), ITS2 (508 bp), lsrDNA (3924 bp), and a 3′ ETS (32 bp). A total of 44 SSRs were identified. These newly developed genomic resources are most relevant for improving the understanding of biology, developing conservation plans, and managing the fishery of the iconic *N. pectoralis*.

## 1. Introduction

**Figure 1:**
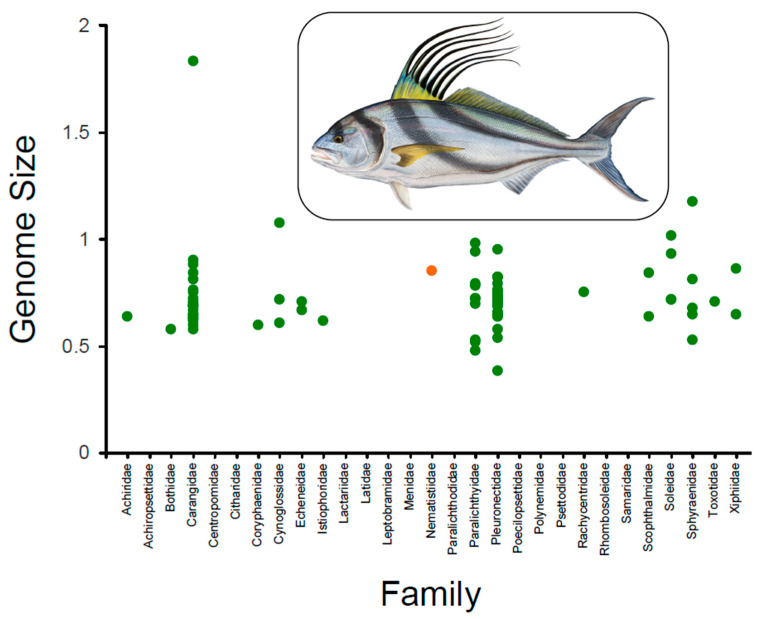
Genome size estimation (Gigabases) using a *k*-mer approach in the ‘Pez Gallo’ or the Roosterfish, *Nemastistius pectoralis* (orange dot), and genome size estimates for other species belonging to different families in the Carangiformes order sensu Girard et al. [8]. Genome size estimates retrieved from www.genomesize.com (accessed on 5 April 2021). The inset at the top depicts a specimen of *N. pectoralis* (art credit: Flick Ford).

Among vertebrates, one of the most speciose chordate clades, the marine and freshwater bony fishes (superclass Pisces; class Actinopterygii) [1], exhibit remarkable disparity in terms of morphology, physiology, behavior, and ecology [2]. Among them, the ‘Pez Gallo’ or the Roosterfish, *Nematistius pectoralis* Gill, 1862, is an ecologically relevant species in shallow (0–20 m) soft-bottom marine and estuarine environments [3,4,5,6] and a target of a lucrative sport fishery in the Central Eastern Pacific Ocean [3,7]. The Roosterfish is an iconic and highly appreciated ‘trophy fish’ by anglers, in large part due to its impressive seven-stranded dorsal fin that resembles a rooster’s comb and from which it receives its name (Figure 1).

*N. pectoralis* inhabits from the northern Baja California and the Sea of Cortez, Mexico in North America, to San Lorenzo, Peru, in South America [6,9]. It is also present in the Galapagos archipelago and Malpelo Island [6,9]. The life history of *N. pectoralis* is poorly known [3,4,5,7,10]. *N. pectoralis* can grow up to 191 cm and weigh 52 kg, and together with various other large fishes, e.g., albacore or swordfish (*Xiphias gladius*), marlins (*Makaira* spp.), and dorado (*Coryphaena hippurus*)*,* among others, it is targeted by a multi-million-dollar recreational fishing industry across most of their geographic range, but especially in Baja California Sur, Mexico and Nicoya, and the Osa Peninsula, Costa Rica [6,7]. According to the International Union for Conservation of Nature (IUCN), the Roosterfish is assessed globally as Data Deficient (www.iucnredlist.org accessed on 28 September 2021). Despite the commercial value and its ecological relevance, no genomic resources exist for this species. One of the few genetic resources available for the Roosterfish is a set of 16 microsatellite primers recently isolated and characterized by Molina-Quirós and Hernández-Muñoz [11]. Thus, the development of additional genetic and genomic resources in the Roosterfish *N. pectoralis* is most relevant as they will provide an opportunity to continue improving the understanding of the biology of this species while also aiding in its fishery management and conservation.

This study forms a part of a broad effort aimed at developing genomic resources in *N. pectoralis* and other species targeted by the sportfishing industry in the Central Eastern Pacific Ocean. Specifically, using a low-coverage short-read next-generation sequencing approach, this study, for the first time: estimated the nuclear genome size, estimated single-copy and low-copy number gene genome content, discovered, annotated, and characterized nuclear repetitive elements, and assembled and annotated the 45S rRNA DNA operon. A set of microsatellites or short sequence repeats (SSRs) was also discovered. All these newly developed resources are most relevant for improving the understanding of biology, developing conservation plans, and managing the fishery of the iconic *N. pectoralis*.

## 2. Material and Methods

### 2.1. Sampling and DNA Extraction

Field collection was approved by CONAGEBIO (permit number: R-CM-VERITAS-001- 2018-OT-CONAGEBIO) and INCOPESCA (permit number: INCOPESCA-CPI-001- 05-2018). One individual of *N. pectoralis* was captured by fishermen during an expedition to the locality of Paquera (9.490091° N, 84.51915° W), Nicoya Peninsula, Pacific Coast of Costa Rica (Table 1). The total genomic DNA was extracted from the muscle tissue of the captured specimen using the Promega Wizard™ SV Genomic DNA Purification Kit (Promega Inc, Madison, WI, USA) following the manufacturer’s protocol. Extracted DNA was then shipped to the Savannah River Ecology Laboratory, at the University of Georgia, for next-generation sequencing.

### 2.2. Library Preparation and Sequencing

An Illumina paired-end (PE) shotgun library was prepared using the standard protocol of the Nextera^TM^ DNA Sample Preparation Kit (Epicentre^®^, San Diego, CA, USA), and sequenced in an Illumina HiSeq-2500^®^ platform (Illumina, San Diego, CA, USA) using a 2 × 300 cycle (insert size = 150). A total of 14,512,060 million pairs of reads were generated (corresponding to a ~3x genome coverage per nucleotide) and are available in the short-read archive (SRA) repository (Bioproject ID: PRJNA772885; Biosample accession: SAMN22417937; SRA accession: SRR16493600) at GenBank.

### 2.3. Genome Size Estimation in Nematistius pectoralis

Contaminants, low-quality sequences (Phred scores < 20), and Illumina adapters were removed using the software fastp v.0.20.1 [12] with default parameters, leaving 13,992,934 high-quality reads. The totality of these paired reads was used to estimate genome size by counting *k*-mers with a word size equal to 21 in the software Jellyfish-2 v.2.3.0 [13]. The *k*-mer frequency distribution was then processed with the program REPeat SPECTra Estimation (RESPECT) v.1.0.0 [14].

### 2.4. Repetitive Elements in the Nuclear Genome of Nematistius pectoralis

The discovery, annotation, and quantification of the repetitive elements in the genome of *N. pectoralis* were conducted as described in Baeza [15], using a portion of the reads automatically selected by the pipeline RepeatExplorer v.2.3.8 [16] available in the Galaxy platform (http://repeatexplorer.org/, accessed on 10 April 2021). RepeatExplorer efficiently analyzed the repeat composition and abundance of plant and animal genomes with low-coverage Illumina PE sequences [16,17]. RepeatExplorer started with an all-to-all sequence comparison to find similar pairs of reads (90% sequence similarity spanning at least 55% of the read length) and built graph-based clusters of overlapping reads that represented different individual families of repetitive elements. Each of the identified repetitive element clusters was further classified when annotated using an internal database. Within each cluster (family of repetitive elements), the reads were assembled into contigs using the program CAP3 [18] and annotated using the Metazoa version 3.0 repeat dataset included in the package. All other parameters in RepeatExplorer were set to default values. The genome proportion of each identified repetitive element cluster was calculated as the percentage of reads [16].

### 2.5. Nuclear Ribosomal Operon in Nematistius pectoralis

The nuclear ribosomal operon that codes for the large and small nuclear rRNA genes (18S or ssrDNA, 28S or lsrDNA), the 5.8S rDNA gene, the two internal transcribed spacers (ITS1 and ITS2), and the two external transcribed spacers (5′ ETS and 3′ ETS) in the genome of *N. pectoralis* was retrieved after assembling contigs using all the reads with the program SPAdes v3.11, [19] as implemented in the pipeline Shovill (https://github.com/tseemann/shovill, accessed on 2 April 2021). We used the software Bandage [20] to visualize the assembly graph produced by Shovill. Considering that the nuclear ribosomal operon is a repetitive element [21], we predicted that an unusually high (above average) coverage contig of ~ 7–10 kbp in length would be observed when visually inspecting the assembly graph produced by Shovill, if this tool successfully assembled the 45S rRNA DNA operon in *N. pectoralis*. Each observed contig >7 kbp (*n* = 3) in length was blasted against the non-redundant (nr) nucleotide NCBI database as well as Dfam [22] and Rfam [23]. Contigs that did not match fish ribosomal sequences with *E*-values < 1 × 10^–6^ were discarded (*n* = 2). The remaining contig, and another 2 contigs > 1000 bp assembled by CAP3 that were annotated as nuclear repetitive ribosomal DNA by RepeatExplorer, were aligned with Muscle [24] with the default parameters as implemented in MEGA [25]. The assembly was curated manually. The exact coding positions of the 18S and 28S nuclear rDNAs and the boundaries of the 5′ and 3′ ETSs were determined using RNAmmer in the RNAmmer v1.2 Server (http://www.cbs.dtu.dk/services/RNAmmer/, accessed on 29 March 2021) with default parameters [26]. The exact coding positions of the 5.8S nuclear rDNA and the boundaries of the ITS1 and ITS2 were determined using ITSx v.1.1b1 [27].

### 2.6. Microsatellite Discovery in Nematistius pectoralis

Simple sequence repeats (SSRs) in the genome of the Roosterfish were identified using the pipeline Pal_finder v0.02.04.08, as implemented in the platform Galaxy (https://palfinder.ls. manchester.ac.uk, accessed on 28 September 2021) [28]. The pipeline first scanned all short reads for the existence of SSRs (dinucleotide, trinucleotide, tetranucleotide, pentanucleotide, and hexanucleotide motif repeats). Next, PCR primers were developed using default parameters in the software Primer3 [29]. We applied the default settings and the most stringent filtering options in the pal_filter to select optimal SSR loci; only loci with ‘perfect’ motifs ranked by motif size and with designed primers were included. The Loci, where the primer sequences occurred more than once in the set of reads, were excluded. A minimum of 5 repeats was requested for the program pal_finder to select 2-mer SSRs and a minimum of 6 repeats to select SSRs with 3, 4, 5, and 6 repeat motifs. Lastly, the software PANDAseq [30] was used to assemble paired-end reads and confirm that the primer sequences were present in the assembly based on the available reads.

## 3. Results and Discussion

### 3.1. Genome Size Estimation in Nematistius pectoralis

The average haploid genome size of *N. pectoralis* estimated using a *k*-mer approach was 816.04 Mbp, with a unique genome content (63%). Sequenced fish genomes vary in size from 342 Mbp in *Tetraodon nigroviridis* to 2.967 Gbp in *Salmo salar* [31]. Genome size varies moderately in fish belonging to the Carangiformes order sensu [8], from 0.39 Gbp in *Pleuronectes platessus* (fam. Pleuronectidae) to 1.87 Gbp in the Florida pompano (*Trachinotus carolinus*, fam. Carangidae) (Animal Genome Size Database (http://www.genomesize.com/, accessed on 4 May 2021)—Gregory, [32] (consulted on 30 March 2021)) (Figure 1). We note, however, that genome size estimates in the database above are based on C-values determined either through flow cytometry or Feulgen densitometry. Genome size in species of the Carangiformes order estimated using a *k*-mer approach found in the recent literature ranges from 544.2 Mbp in the slender sharksucker (*Echeneis naucrates*) to 716.4 Mbp in *Seriola lalandi dorsalis* see Table 3 in [33]. The estimated genome size of *N. pectoralis* is well within the range observed in the Carangiformes order. This moderately large genome size, combined with the relatively high abundance of repetitive elements (27%), suggests that a combination of both short and long-reads (i.e., Oxford Nanopore Technology and Pacific Biosciences) will likely be needed for the assembly of a high-quality genome in this species.

### 3.2. Repetitive Elements in the Nuclear Genome of Nematistius pectoralis

The RepeatExplorer pipeline analyzed a sub-sample of 1,171,363 reads, 1,168,660 of which were contained in 530,345 clusters (families of repetitive elements). The proportion of reads contained in the top 60 clusters that represented the most abundant repetitive elements in the genome of *N. pectoralis* was relatively low (3.9%). Significantly, a large portion of the top repetitive element families (*n* = 43 clusters, 24,757 reads) were reported as ‘unclassified’ by RepeatExplorer, given that they could not be assigned to known repeat families. The above is in line with previous studies exploring the repeatome in other fishes and suggests that abundant new repetitive elements will be discovered by future studies focusing on the repetitive elements of *N. pectoralis* and other distant and closely related fishes [31] and references therein. Taking into account only annotated clusters (*n* = 17), the most common repetitive elements were classified as Satellite DNA (*n* = 7 clusters, 12,917 reads) which were considerably more abundant than Class I-Long Interspersed Nuclear Element (LINE) (*n* = 3 clusters, 5172 reads) and Class I-LTR Retrovirus elements (*n* = 1 cluster, 690 reads). Six clusters were classified as 45S rRNA DNA (18S (*n* = 5 clusters, 1122 reads) and 28S (*n* = 1 cluster, 798 reads).

Overall, this analysis revealed that a large part of the annotated repeat elements represents Satellite DNA in the genome of *N. pectoralis*. This dominance of Satellite DNA in the nuclear genome of *N. pectoralis* is in line with that reported in other fishes and supports the notion that marine bony fishes harbor more tandem repeats than freshwater species [31]. Only recently, repetitive element profiles have been explored in fish due to the increasing availability of sequenced fish genomes [31].

### 3.3. Nuclear Ribosomal Operon in Nematistius pectoralis

**Figure 2:**
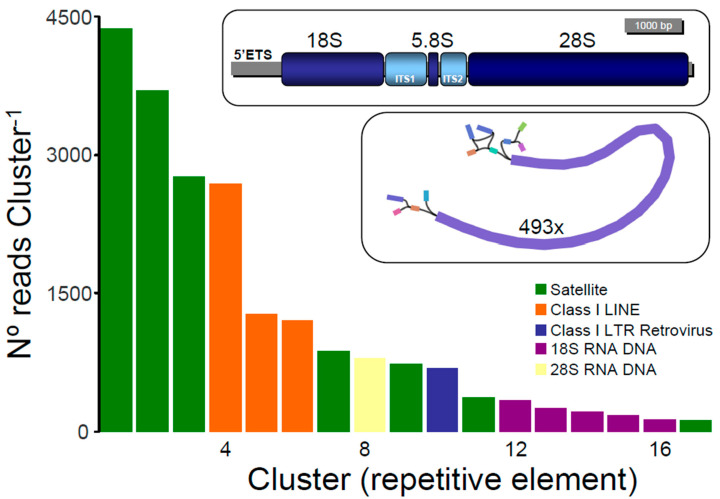
Size distribution (N° = number) and repeat composition of annotated clusters generated by similarity-based partitioning in the Roosterfish. Bars are colored according to the type of repeat present in the cluster, as determined by the similarity search in RepeatExplorer2. The inset in the top right depicts the 45S ribosomal operon in *Nematistius pectoralis*. The second inset underneath the first inset shows the single 45S rRNA DNA unit contig assembled with the program Shovill.

The strategy employed in this study permitted reconstructing the entire 45S rRNA DNA operon in *N. pectoralis* (Supplementary Materials File S1). A single contig assembled in Shovill with unusually high coverage of (493×) 8129 bp in length was determined to be the 45S rRNA DNA operon of *N. pectoralis* after examining the assembly graph in the software Bandage and contigs blasts against the nr NCBI, the Dfam, and the Rfam databases (all circular contigs matched the 45S rRNA DNA operon of Teleostei representatives available in GenBank with *e*-values << 1 × 10^−10^). The nuclear ribosomal operon is comprised of, in the following order: a 5′ ETS (length = 948 bp (likely partial sequence)), ssrDNA (1835 bp, full sequence (fs)), ITS1 (724 bp (fs)), a 5.8S rDNA (158 bp (fs)), ITS2 (508 bp (fs)), lsrDNA (3924 bp (fs)), and a 3′ ETS (32 bp (partial sequence)), as demonstrated in Figure 2.

The organization of the newly described 45S rRNA DNA unit in the Roosterfish is identical to that described for other fishes [34]. The newly described genomic organization of the ribosomal operon will serve as the base for species-specific marker discovery [35]. Furthermore, this new information will also facilitate the development of new low-pass sequencing + gene-targeted phylogenomic approaches as an alternative or addition to, for example, ultraconserved elements [36] and anchored hybrid enrichment [37] to study the phylogenetic relationships among representatives of the Carangiformes order.

### 3.4. Microsatellite Discovery in Nematistius pectoralis

A total of 44 SSR primer pairs N = 32, 9, and 3 for 2mer, 3mer, and 5mer SSRs motifs, respectively, were identified using the most stringent filtering options for finding SSRs in pal_finder (Table 2). The software pal_finder did not retrieve SSRs with 4mer and 6mer motifs.

Studies exploring population genetics in *N. pectoralis* are lacking. Recently, Molina-Quirós and Hernández-Muñoz, [11] developed the first set of microsatellites (*n* = 16 SSR loci) for this species. Future studies combining mitochondrial protein-coding genes or whole mitochondrial genomes (under development) and a subset of previously or newly identified SSRs (after further development) can be used to assess the population genomic structure and connectivity in *N. pectoralis* across its entire range of distribution in the Central Eastern Pacific Ocean.

## 4. Conclusions

This study developed, for the first time, genomic resources for the iconic ‘trophy’ gamefish, Roosterfish *N. pectoralis*, an ecologically important species and a target of a most lucrative recreational fishery in the Central Eastern Pacific Ocean. Using low-pass short-read Illumina sequencing, the genome size and single/low-copy gene content were estimated, nuclear repetitive elements were identified and partially classified and quantified, and the ribosomal RNA operon was assembled and annotated. A large set of SSRs was also detected. This information will contribute to a better understanding of the meta-population genomic structure and connectivity and the genomic mechanisms involved in the acclimatization and adaptation to local and global climate change in *N. pectoralis*.

## Figures and Tables

**Table 1:** MlxS descriptors of the study.

Item	Description
Submitted_to_insdc	Yes (SRA)
Investigation_type	Eukaryote
Project_name	*Nematistius pectoralis* WGS
Geo_loc_name	Paquera, Costa Rica
Lat_lon	9.490091° N, 84.51915° W
Depth	10 m
Alt_elev	0 m
Collection_date	2016-10-06
Collected_by	José Luis Molina-Quirós
Env_biome	Seawater (ENVO:00002149)
Env_feature	Bay (ENVO:00000032)
Env_material	Seawater (ENVO:00002149)
Env_package	Water
Temp	NA
Salinity	NA
Sequencing method	Illumina HiSeq2000
Assembly method	Shovill v.1.0.0

**Table 2:** Microsatellites in the Roosterfish.

Motifs(Bases)	Forward Primer Name	Forward Primer Sequence	Reverse Primer Name	Reverse Primer Sequence
AAGAG(50)	rooster_F1	TGACCAATCGTCTCGTCTCG	rooster_R1	ACTGCTGGGCAGCTTTTAGC
AAGAG(40)	rooster_F2	TGTGTATTTTATTTTCCAATACATGTAGGC	rooster_R2	CTGTGTGTGTCCCTTGCTCG
AAAAG(50)	rooster_F3	CAGACAGTGTTGGGTACACCG	rooster_R3	CCTGTGCTGTTTCTTGCTGC
TGC(18)	rooster_F4	AGGTGAGAGTCGCTGCTGG	rooster_R4	TCAAACCTCCTCAGCATCCC
TTG(15)	rooster_F5	CATGAAGCTGGTTAACTGTGCC	rooster_R5	ACAGACAACGGCAACTGTGG
TGG(21)	rooster_F6	ACACAGGGCTCTGACAAGGG	rooster_R6	CCATACAAGCGATGTCTGGC
TGC(15)	rooster_F7	ACAACCTTTCCCCTCAGTGC	rooster_R7	GAGCTGGCAGGATCTGTGG
AGG(18)	rooster_F8	ATAGTTGCCCGCCAAACG	rooster_R8	CGGCTTCAGCTTCCTACTCC
ATT(21)	rooster_F9	TACATGGGACACATCACCCG	rooster_R9	TTGTGTTGGGATTCAGTGCC
TGG(15)	rooster_F10	TTAAAGCAACGCTGCTGACG	rooster_R10	ACCGAATAGGTTGTTGTTGGG
AAG(15)	rooster_F11	TCGGTGCTGGTTACCATTAGG	rooster_R11	CAAACGTTCCACCCAGAAGG
AAC(18)	rooster_F12	AGGATGGGGATTCCTTCACC	rooster_R12	GGGCAATCTCTTAAGCTGCC
AC(18)	rooster_F13	TTCTGGAGTTTACTGGGGTTCC	rooster_R13	AGGTGACCTGGAAGCAAAGG
AG(16)	rooster_F14	AGACCAGGCTGTCTCTCTCTCC	rooster_R14	GCTAATTGAAATGCCGCTGG
AG(12)	rooster_F15	AGTGAGTTTGCGTGATTGGG	rooster_R15	CATGGAAACCTTGCCAGAGC
TG(12)	rooster_F16	CCCTCCAGGGAATTTGTACG	rooster_R16	CTGACAGCTAGCCCAGGTCC
TG(40)	rooster_F17	CATGTATATGCCATTTTATGTCTGTCC	rooster_R17	TCGGTGGTTGTTGTCTTTTCC
TG(22)	rooster_F18	CAGTCTAGCACCATTCTGGGG	rooster_R18	TTCTGCTACTTGCTGAGCCG
AC(12)	rooster_F19	GGCAGCTGGAGTGAAAGTAAGG	rooster_R19	TGCAGAAAGTAGTGTGGACTTGG
TG(14)	rooster_F20	CTTCGAGGAGGCCTGTTACC	rooster_R20	TGGCCTAAATACAGGCTTGG
AT(14)	rooster_F21	GTGCTGGTTTAAAGGCAACG	rooster_R21	GCAGCTCATCGAAAGAATGC
AC(20)	rooster_F22	TAGCGATGGCACTTTCATGG	rooster_R22	GGCAGAGATCATAATTGCTGTGG
AC(20)	rooster_F23	TGTGCGTCTCTTGTGTCTGG	rooster_R23	AGATTAAGAGAGCGTGTGAGCG
AG(12)	rooster_F24	CCATCTCTCGCCAATTCTCC	rooster_R24	TGTTGCAATTTGATAGTCTGGC
AG(18)	rooster_F25	AAGATTCACTTTGCTTCAAGGC	rooster_R25	TAACGAGTATCCAGAGCGGG
AG(12)	rooster_F26	CAGCAGGGTCTGAAGCAAGC	rooster_R26	CTGCCCTTCCTGCTGTTACC
TG(24)	rooster_F27	CTCATGGGAAGAGACAAGTAGTCC	rooster_R27	GCCTCCTGTTGTAAGCCTGC
AC(12)	rooster_F28	TTAAACCATCCTTGAGTGTGTGG	rooster_R28	TCCCAAAGCAGATACCCACC
TG(24)	rooster_F29	TGGGCATATTTTGGTTAACGG	rooster_R29	AGTGGTTGTCCTCATCACCG
AC(18)	rooster_F30	CGAAAAGGTCCTTGACGAGC	rooster_R30	ACATGTCGCAAAGGAGAGGG
TG(50)	rooster_F31	GTTGCATGGCAGCTCTATCG	rooster_R31	AACCCACCCCAGCAAAGC
TG(16)	rooster_F32	GAAAACACGAGGGCAGTACG	rooster_R32	CCACAGCAGAAACACAATGG
AT(12)	rooster_F33	TTTGATACAGGATTTAGGTGCCC	rooster_R33	GGAGAGGAGCGTAGGAATGG
AC(54)	rooster_F34	TCGAAATAAGGGAGAGAGCAGC	rooster_R34	GGAACAGCTTTGGAGGATGG
AC(20)	rooster_F35	CACAATGCATTAGGACCTCCG	rooster_R35	AGAAGGAGAGATAGCCCCGC
TG(18)	rooster_F36	AGACACCAGCACACACGTCC	rooster_R36	GTTGTCCAAACACCAGCAGC
TG(12)	rooster_F37	GAAATCAATAGCGGATTCGACC	rooster_R37	ACCATGATATTTCTGCCGGG
TG(12)	rooster_F38	GATAAATGCGCCACACTTGG	rooster_R38	GCATGTAAGAGCAGGGTTGC
AC(36)	rooster_F39	CTTTGAGTCTTACTTTTATAATGTGCTCC	rooster_R39	CTTTGGAAAAGGGACAACCG
TG(12)	rooster_F40	CGGCAGAAATGTGTTTGAGC	rooster_R40	CCACATAGCCTTCATTTCACTCC
TG(14)	rooster_F41	ACACCAACCCACCCACTAGC	rooster_R41	TGAATGCGTGGATGGTATCG
AG(12)	rooster_F42	ACAGAGCAGCCTGTATGGGG	rooster_R42	CTGAGCCAGAGAAAGGAGGG
AG(12)	rooster_F43	CCCAGATCCTTTCATCCAGC	rooster_R43	AATCTCACCGATGCGTTTCC
TG(32)	rooster_F44	ATGATGATGAACGCAGAGGG	rooster_R44	GAGCCACTAGCCAGTCCTGC

## Data Availability

DNA-seq data have been deposited in the NCBI Sequence Read Archive (SRA) under Bioproject ID: PRJNA772885, Biosample accession: SAMN22417937, and SRA accession: SRR16493600.

## Supplementary Materials

The following are available online at https://www.mdpi.com/article/10.3390/genes12111710/s1, File S1: 45S rRNA DNA operon in *Nematistius pectoralis*.
